# Supplementary material for: Observation of an unpaired photonic Dirac point
Source: Nat Commun. 2020 Apr 20;11:1873. doi: 10.1038/s41467-020-15801-z (PMC7171084; doi:10.1038/s41467-020-15801-z)
Supplement: Supplementary file 1 — Supplementary Information [file 41467_2020_15801_MOESM1_ESM.pdf]

Supplementary Information

Observation of an unpaired photonic Dirac point

Liu *et al.*

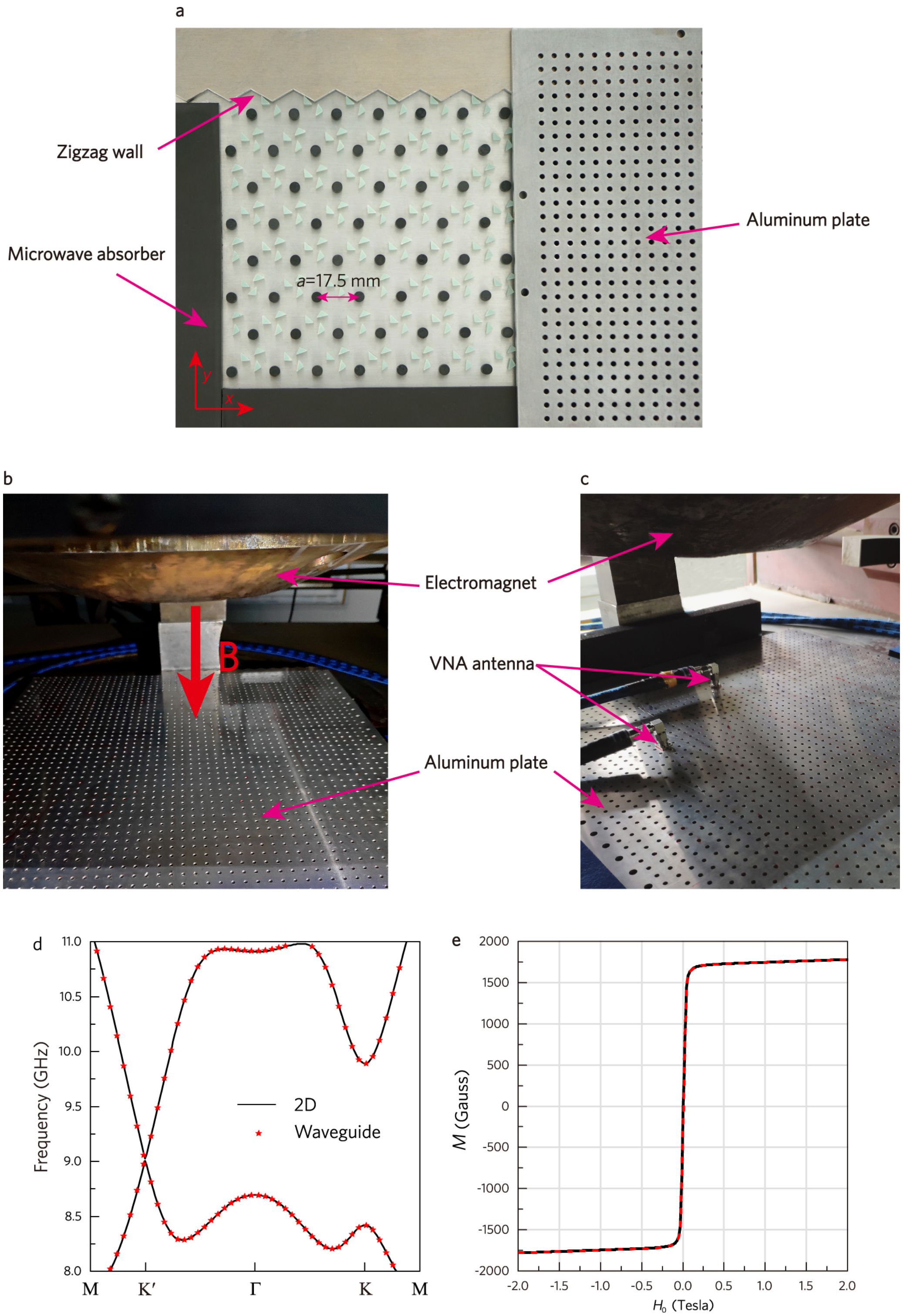

Supplementary Figure 1 | Experimental details. (a) Photography of the sample arrangements. **(b)-(c)** Field-mapping setups. The *z*-oriented external static magnetic field is generated by a large electromagnet; the spatial non-uniformity of the magnetic field is less than 2% across the sample. Pixeled holes with a diameter 1.8 mm and a period of 8 mm are drilled through the top aluminum plate. Such holes facilitate the experimental measurement. Two identical dipole

antennas are employed as the source and receiver antennas, respectively. Both antennas are inserted into the waveguide via the holes, and connected to a vector network analyzer (R&S ZNB20) to collect the transmissions. Field distributions are measured by mapping the local field at the different holes one by one. The four sides of the samples are coverved with microwave absorbers. **(d)** Bandstructure of the PhC sample with θ=12.9° and B=0.4 Tesla. Solid black lines represent the results simulated in 2D; red star denotes the practical structures placed in a waveguide with pixeled holes drilled on top plate. Note that as the holes are at deep subwavelength scale, which have negligible effect on the electromagnetic-wave mode inside the parallel waveguide around 9.0 GHz. **(e)** Measured hysteresis loop of the gyromagnetic YIG ferrite. The magnetic hysteresis loop measured by Vibrating Sample Magnetometer implies excellent soft magnetic properties of our YIG ferrite, i.e. magnetically saturated at small external field ~0.05 Tesla and possessing negligible remanence ~10 Gauss.

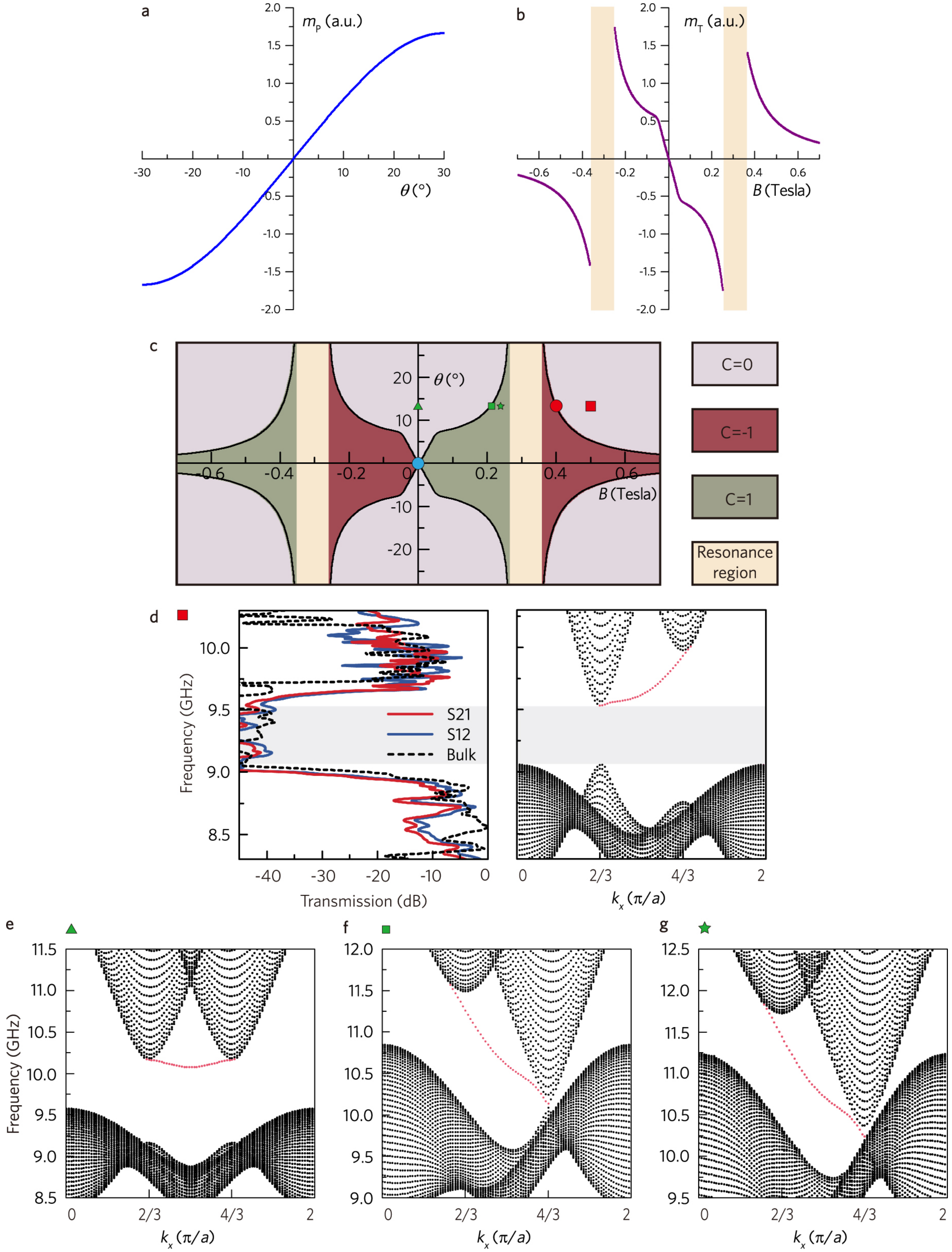

Supplementary Figure 2 | Haldane-type Phase diagram. (a)-(b) The relative mass terms m_P and m_T as functions of rotation angle θ and magnetic field B, respectively. The m_P and m_T are normalized to $m_{\mathrm{P}(\theta=12.9^\circ)}$ and

$m_{\mathrm{T}(B=0.4\ \mathrm{Tesla})}$, respectively. The yellow shadows indicate the strong ferromagnetic resonance region. **(c)** The Haldane-type phase diagram. The black lines denote the boundaries between the Chern insulator and the topologically trivial insulator. The three crucial states, the unpaired Dirac point (θ=12.9°, B=0.4 Tesla, red circle), the trivial insulator (θ=12.9°, B=0.5 Tesla, red square), and the paired Dirac points (θ=0°, B=0 Tesla, blue circle), are experimentally studied. For comparison, three states (θ=12.9°, B=0 Tesla, green triangle; θ=12.9°, B=0.2 Tesla, green square; θ=12.9°, B=0.22 Tesla, green star) are numerically investigated. **(d)** Experimental bulk/edge transmission and numerical band diagram for the PhC at the state (θ=12.9°, B=0.5 Tesla, red square). No edge state exists, indicating a topologically trivial state. **(e)-(g)** Numerical band diagrams for the PhC in the comparison states. The black/red circles represent the projected bulk/edge states localized along the upper zigzag boundary. Note that domain walls are included for all the numerical studies. This state is not a single-mode state. It is difficult to be measured in experiments and hence limited for practical applications.

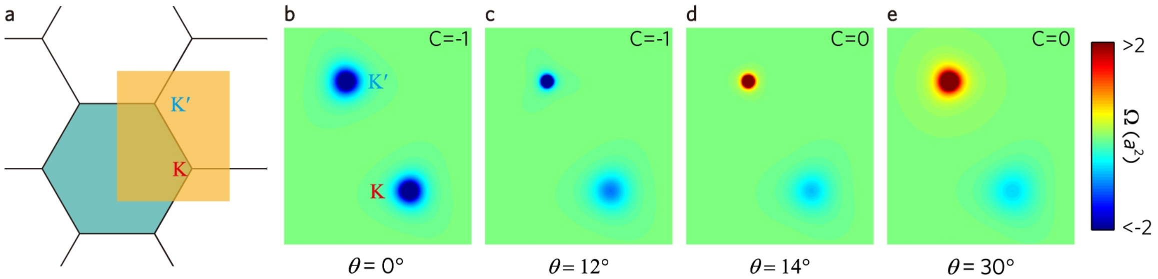

Supplementary Figure 3 | Berry curvature of the second band in momentum space. (a) Berry curvature in the orange shadowed region $0 \le k_x \le 2\pi/a$ and $-\sqrt{3}\pi/3a \le k_y \le \sqrt{3}\pi/a$. **(b)-(e)** Numerical Berry curvatures for PhCs with θ=0°, 12°, 14°, and 30°, respectively. For θ=0°, the Berry curvatures near K and K′ have the same profiles, and the integrals around each valley is close to $-\pi$, which gives a total Chern number of –1. Upon increasing θ, the integral of the Berry curvature around K′ changes from $-\pi$ to π (this occurs abruptly between θ=12° and 14°. Thereafter, the integral of the Berry curvature vanishes and the Chern number is 0.

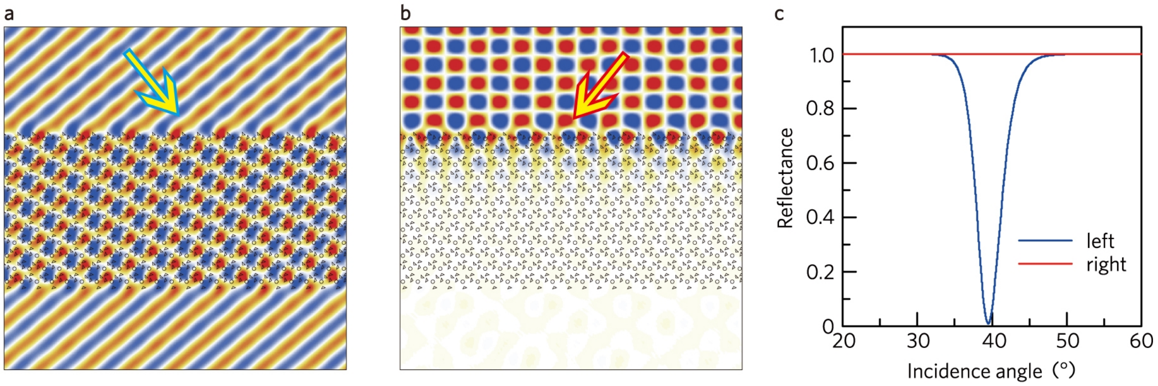

Supplementary Figure 4 | Non-reciprocal reflections of plane waves. (a) The incident plane wave from the left-upper region with an incidence angle α=39.1° (indicated by the blue arrow) can couple into the PhC slab (θ=12.9° and B=0.4 Tesla). **(b)** The incident plane wave from the right-upper region with an incidence angle α (indicated by the red arrow) is reflected. **(c)** Reflectance as a function of the incidence angle of the left- and right-input plane waves. The sharp contrast between right/left reflections reaching 100% versus 0% has been achieved.
